# A reverse factual analysis of the association between smoking and memory decline in China

**DOI:** 10.1186/s12939-016-0417-6

**Published:** 2016-08-22

**Authors:** Yingying Yi, Ying Liang, Guoqiang Rui

**Affiliations:** 1 School of Economics, Nanjing University of Posts and Telecommunications, Nanjing, 210023 Jiangsu Province People’s Republic of China; 2 Department of Social Work and Social Policy, School of Social and Behavioral Sciences, Heren Charity Academy, Nanjing University, Nanjing, 210023 Jiangsu Province People’s Republic of China; 3 ShiLiang School of Law, Changzhou University, Changzhou, 213164 Jiangsu Province People’s Republic of China

**Keywords:** Smoking, Memory decline, Reverse factual analysis, Propensity score matching

## Abstract

**Background:**

Whether smoking accelerates memory recession has been a topic of significant research. However, randomised controlled trials are not easy to carry out, and does not comply with the ethics of research. And observation method which based on the most readily observed data is easy to draw the wrong conclusions without adjustment. The memory difference between smokers and non-smokers may not really represent the real differences between their memories.

**Methods:**

In response to these limitations, we adopt propensity score method to match the samples and solve the estimated selection bias and confounding bias on elderlies aged 60 years and over based on Chinese Longitudinal Healthy Longevity Survey (2011) data. The respondents are divided into non-smokers, people who used to smoke but not now, and people who used to smoke and still now. To balance the similarity between different groups on their propensity score weighted distributions of pretreatment covariates, we use generalized boosted models to estimate the multiply treatment propensity scores.

**Results:**

The results show that compared with non-smokers, people who used to smoke and still now respectively have a decrease 0.0283, 0.0735, 0.0091 on self-evaluation memory, daily living activities, and cognitive function. People who used to smoke but not now have a decrease 0.0224 on daily living activities, while have an increase 0.0054 and 0.0104 on self-evaluation memory, and cognitive function.

**Conclusion:**

The PSM has considerable utility to control pre-treatment imbalances on observed covariates in non-randomised or observational data.

## Background

China is the world’s largest producer and consumer of tobacco [1]. It has 350 million smokers and accounts for 37 % of global tobacco production [2]. The increasing number of smokers and consequent effects has gained public attention [3]. Many scholars identified that smoking can be related to many things, e.g. substance abuse or dependence, increased work time, social isolation, negative life events, family breakdown, child abuse, behavioural problems, family history of smoking and anxiety, etc. [4–9]. Even passive smoking also influences all life stages of Chinese elderly, including the risk of depression, daily life ability impairment, the odds of self-reported chronic diseases, and the impacts of cognitive function on social participation [10].

In recent years, researchers pay more attention to the negative impacts of smoking on working memory [11, 12]. A longitudinal study for eight long-term smokers found the decline of their memory, cognitive function, and attention ability was closely related to smoking [13]. Compared with non-smokers, smokers have weaker performance in cognition and memory, and, in the long run, are more likely to suffer from depression and anxiety [14–18]. For adolescents, smoking does more serious damage to youths’ working memories [19, 20]. For smokers between ages 43 and 53 who smoke more than 20 cigarettes a day, memory recession is faster than in youths [21]. For elderly smokers who smoke or smoked, compared to those who have never smoked, had more severely declining memory and cognitive functions as well as larger risk for Alzheimer’s disease [22–24]. The reason is that the harmful substances in tobacco or nicotine negatively impact people’s sleep quality, and consequently damage memory and cognitive functions [25]. Smokers’ working memory ability and cognitive efficiency are significantly lower than non-smokers, so people should pay attention to smoking and memory impairment [26]. However, some researchers find that working memory and ability of the short-term smokers were improved compared to that of the non-smokers [27].

Along with aging, memory is also affected. Whether smoking accelerates memory recession has been a topic of significant research. In some studies, scholars used randomised controlled trials, so the behaviours and results of smokers and non-smokers could be easily observed. Randomised controlled trials need to recruit a large number of participants, who are then randomly assigned to smoking and non-smoking groups. Nevertheless, this type of experiment is not easy to carry out, and does not comply with the ethics of research. In this case, observation is the most appropriate method. However, based on the most readily observed data, it is easy to draw the wrong conclusions without adjustment. For example, when comparing the best memory of the smoking group and the poorest memory of the non-smoking group, we would come to the conclusion that smoking is harmless to memory. The reason is that observation study does not adopt randomised grouping, which weakens the influence of confounding variables in the treatment and control groups. Therefore, it is easy to cause systematic bias. The Propensity Score Matching (PSM) method can solve this problem and eliminate interference factors between the two groups. This research study employed the PSM method to match samples, using the propensity score to control covariates, and solve the estimation bias caused by self-selectivity.

## Methods

### Sample

Research data for this study came from the latest survey data of the Chinese Longitudinal Healthy Longevity Survey (CLHLS) from 2011. The CLHLS conducted face-to-face interviews in 1998, 2002, 2005, 2008, and 2011, respectively, using internationally compatible questionnaires. The survey design investigated each centenarian in the sampled counties based on the voluntary principle, focusing on the oldest, i.e. ages 80 and older, from 631 counties of 22provinces in China.^1^ In order to yield a comparable sample, the CLHLS included young elderlies aged 65–79 and their aged 35–64 adult children from 2002 onward. Questionnaires included basic conditions of respondents as well as information on their social, economic backgrounds and family structures, as well as respondent self-evaluation on health status and quality of life, life style, disease, health and other detailed information. The survey project was supported by Demographic Analysis of Health Longevity in China and Duke University in 1998, the United Nations Population Fund (UNFPA) and China Social Sciences Foundation in 2002, and the China Natural Sciences Foundation and Hong Kong Research Grants Council in 2004. The CLHLS is currently the most representative micro-panel data related to elderly health, and has the largest global sample of centenarians to a report in *Science*. Related survey data presents high quality in terms of sample loss, accuracy of respondent age, and reliability and validity of main variables [28, 29].

In China, the age benchmark of sixty years is usually referred to as ‘a cycle of sixty years’. China is located in the Asia Pacific region, which generally considers 60 years and older as elderly; the normal retirement age of this region is 60 years old. Therefore, in this paper, the study objects are elderly born in 1951 or before and reached 60 years and above in 2011.

### Measures

In measuring the memory status of the elderly, this research study used multiple indicators to represent the different dimensions of memory in order to analyse the influence of smoking on people’s memory.

#### Self-evaluation memory

Self-evaluation memory is a comprehensive measurement index, including respondents’ subjective and objective memory status.

Survey question: ‘What do you think about your own memory?’

Response coding: ‘excellent’, ‘very good’, ‘good’ = 1; ‘general’, ‘not good’ = 0.

#### Activities of daily living

Memory aging is seemingly normal for adults. Although it tends to inconvenience the elderly, generally speaking, it does not have a great impact on their working, learning and living. This research asked questions to the elderly to measure their daily living. Questions asked included wearing clothes, bathing, talking, waking up, doing housework, cooking, grocery shopping, taking medicine, etc.

Survey question: ‘Whether health or memory causes difficulty in the completion of daily activities?’

Response coding: ‘not difficult’ = 1 (representing good memory); ‘difficult but could still complete’, ‘difficult and need help’, ‘cannot complete’ = 0 (representing general or bad memory).

As long as one elderly response included ‘difficult but could still complete’, ‘difficult and need help’, ‘cannot complete’, that respondent was defined to have general or bad memory.

#### Cognitive function

The aging process is accompanied by the decline of cognitive function. Cognitive function degradation is often an early symptom of Alzheimer’s disease, brain atrophy, and Parkinson’s disease, which have been difficult problems for the elderly across many countries. The questionnaire drew on the internationally popular Mini-Mental State Examination (MMSE) for respondent orientation skills, immediate recall, delayed recall, structural imitation and calculation ability. Scores range from 0 to 31. Education background also influences MMSE scores [30]. Taking into account the low level of education among the elderly, this research utilised coding reflecting Cui et al. [30]. If respondents did not have a formal education, 18 points or less constituted disabled cognitive function. If respondents were educated 1–6 years, 21 points or less indicated disabled cognitive function. If respondents were educated more than 6 years, 25 points or less qualified as disabled cognitive function.

Response coding: disabled cognitive function = 0; good cognitive function = 1.

### Variables

The core independent variable in this study was respondent smoking behaviour. Respondents were divided into non-smokers, people who used to smoke but not now, and people who used to smoke and still now. In order to investigate smoking effects on the memory of the elderly, some of the major demographic characteristics - i.e. social and economic status, family relationship and support, and lifestyle variables - were controlled. American researchers found that male smokers are weaker than female smokers in memorizing people’s names, and that there is no gender difference in the effects of long-term smoking on memory ability; both males and females suffer memory deficits [31]. If the bad habits of smoking continue with aging, they cause memory disorders [32]. Therefore, demographic variables included in this research were age, gender (male coded as 1, female coded as 0), residence (city coded as 1, rural coded as 0), current marital status (married coded as 1, other – unmarried, divorced, widowed - coded as 0), and memory-related disease diagnosis (yes coded as 1, no coded as 0). Blau and Duncan [33] observed that the level of education was an important measurement index for social and economic status as occupation and income. Given the low education of the elderly and the primary school 6-year completion requirement in China, socioeconomic status variables included years of education (more than 6 years of education coded as 2; 1-6 years coded as 1; not received education coded as 0), employment before 60 years old (had a job coded as 1, other coded as 0), and self-evaluated economic level (middle, more than middle and very high coded as 1; lower and poor coded as 0). Cognitive neuroscientists at Michigan State University found that exercises, especially aerobic exercises, could improve long-term memory. In other words, people who do not exercise may not have very good memory. Researchers at Illinois State University found that older people who often exercise have better memories, and neural activities associated with cognitive activities make them more active and effective. In addition, alcohol paralyses the brain and inhibits nerve cells, which results in torpid reaction and affects the hippocampus in the brain. The hippocampus plays a key role in memory; consequently, alcohol causes declining memory [34]. Therefore, lifestyle variables included regular exercise (yes coded as 1, no coded as 0), and drinking habits (do not drink coded as 2, used to drink but not now coded as 1, regular drinker coded as 0).

### Reverse factual analysis

PSM is a method based on reverse factual analysis applying to biology, and was put forward by Paul Rosenbaum and Donald Rubin in 1983. After the 1990s, the method was also applied in health economics and other social sciences. In observational studies, data bias and confounding variables exist due to various reasons. The basic idea of this method is that when studying the effect of a policy or behaviour, comparing similar treated and controlled groups effectively reduces sample selection biases. Compared with traditional matching methods, PSM simplifies the multi-dimension to one single dimension, which reduces computational difficulty and the large sample size requirement, as well as improves the probability of matching success [35–38]. Propensity score methods do not require modelling the mean for outcomes. Accordingly, this research only used pre-treatment covariates and treatment assignments of study participants to implement propensity score adjustments, which avoided bias from model misspecification [39, 40]. Numerous studies have shown the propensity score method can be extended to multiple treatment cases [41–46]).

In the multiple treatment setting, Generalized Boosted Model (GBM) can capture complex and nonlinear relationships on pre-treatment covariates through an iterative process, finding the propensity score leading to the best balance between treatment and control groups [47]. That is, GBM can deal with continuous and discrete variables.

## Results

### Sample selection and description

Research samples were selected based on the following principles: (1) Samples were selected according to respondent birth year; samples born after 1951 were eliminated; (2) Samples missing variable values were deleted. Ultimately, there were 3311 samples, including 1000 non-smokers, 1881 smokers and 430 respondents who used to smoke but did not anymore. Table 1 shows descriptive statistics of all sample data, and compares the three groups. Table 1 shows the gender distribution was relatively balanced, with 1652 males and 1659 females. There were more males who smoked, 852, accounting for 51.57 % of the subsample. Only 433 males did not smoke, accounting for 26.21 %; 367 males used to smoke but did not anymore, accounting for 22.22 % of the male subsample. For females, only 148 smoked, equivalent to 8.92 %. 1448 females were non-smokers, i.e. 87.28 %. 63 females used to smoke but did not anymore, accounting for 3.80 %. Most respondents lived in rural areas, with 1–6 years of education, and mainly engaged in farming before 60 years of age. Furthermore, most were married, with relatively uniform self-evaluated economic levels, and most did not exercise often. Fewer individuals drank at the time of the survey, and non-smokers always do not drink.

**Table 1:** Sample description

Variable	Value	All samples Mean(S.E.)	Not smoke Mean(S.E.)	Used to smoke but not now Mean(S.E.)	Used to smoke and still now	*P*-value
Age	[60,96]	69.2712 (7.0622)	69.5268 (7.3423)	69.6814 (7.0001)	68.6140 (6.4907)	0.002^**^
Gender	1 male	0.4989 (0.5001)	0.2302 (0.4211)	0.8535 (0.3540)	0.8520 (0.3553)	0.000^***^
	0 female					
Residence	1 city	0.2184 (0.4132)	0.2456 (0.4306)	0.2698 (0.4444)	0.1450 (0.3523)	0.000^***^
	0 rural					
Marriage	1 married	0.6527 (0.4762)	0.5875 (0.4924)	0.7581 (0.4287)	0.7300 (0.4442)	0.000^***^
	0 others					
Years of education	2 more than 6 years	1.0359 (0.8844)	0.8767 (0.8948)	1.3093 (0.8019)	1.2180 (0.8338)	0.000^***^
	1 1–6 years					
	0 never be educated					
Whether had a job before 60 years old	1 had a job	0.1915 (0.3935)	0.1834 (0.3871)	0.2465 (0.4315)	0.1830 (0.3869)	0.008^**^
	0 others					
Self-evaluation economic level	1 middle, more than middle and very high	0.5310 (0.4991)	0.5433 (0.4983)	0.5349 (0.4994)	0.5060 (0.5002)	0.159
	0 lower and poor					
Whether have regular exercises	1 yes	0.3781 (0.4850)	0.3828 (0.4862)	0.3605 (0.4807)	0.3770 (0.4849)	0.688
	0 no					
Whether often drink	2 has not been drinking	1.4400 (0.8458)	1.7326 (0.6418)	1.0930 (0.8906)	1.0390 (0.9383)	0.000^***^
	1 used to drinking but not now					
	0 has been drinking					
Whether have been diagnosed with memory-related disease(yes coded as 1 and no coded as 0)	1 yes	0.0233 (0.1478)	0.0202 (0.1407)	0.0326 (0.1777)	0.0220 (0.1468)	0.293
	0 no					
Self-evaluation memory	1 excellent, very good, good	0.1444 (0.3515)	0.1478 (0.3550)	0.1512 (0.3586)	0.1350 (0.3419)	0.592
	0 general, not good					
Daily living abilities	1 good	0.5630 (0.4961)	0.5529 (0.4973)	0.5372 (0.4992)	0.5930 (0.4915)	0.061^.^
	0 general, not good					
Cognitive function	1 good	0.3328 (0.4713)	0.3721 (0.4835)	0.2651 (0.4419)	0.2880 (0.4531)	0.000^***^
	0 disabled					
Sample size	—	3311	1881	430	1000	--

Note: 10 % significance level; *5 % significance level; **1 % significance level; ***1 % significance level

For all samples, the mean values of self-evaluated memory, daily living abilities, and cognitive function were 0.1444, 0.5630 and 0.3328, respectively. Non-smokers were the best in cognitive function. People who used to smoke but did not anymore were the best in self-evaluated memory. People who smoked were the best in daily living abilities. This research used the F test to find that differences of self-evaluated memory among the three groups were not significant (0.1 % significance), while differences of daily living abilities were 10 % significant.

### Balancing the treated and controlled groups

These three respective elderly groups exhibited good performances in one certain aspect, we could not infer that smoking declines memory. This relationship may have been endogenous, so comparing the three groups directly would lead to estimation bias because residual error may include ‘disease’ factors which are related to smoking but cannot be controlled by observable variables. Therefore, the effects of smoking on memory may be exaggerated or reduced [48]. As a result, this researchused generalized boosted regression to estimate propensity scores and weighting of compared cases to estimate the average treatment effect on the treated group.

The study utilised two methods to assess the balance, or equivalence, established on pre-treatment covariates of the weighted treatment and control groups [47]. One method was to use the effect size or the absolute standardised bias and summarize across variables with the mean; the other method was to use Kolmogorov-Smirnov (KS) statistics to assess balances and summarise using the maximum across variables. 5000 iterations were taken to be the optimal number for minimising the largest of KS statistics. Table 2 shows how well the weights succeeded in manipulating the control group to match or balance characteristics of the two groups. In Table 2, E(Y1|t = 1) and E(Y0|t = 1) respectively represent the treatment means and the control means for each of the covariables, while KS and P are the Kolmogorov-Smirnov test statistic and its associated *p*-value. *P*-value is derived from Monte Carlo simulations for the maximum KS statistic. Thus, a small *p*-value indicates the groups are clearly imbalanced and inconsistent with what should be expected had the groups been formed by random assignment. From Table 2, it is evident balance was achieved after weighting.

**Table 2:** Balance of the treatment and comparison groups

	Var	E(Y1|t = 1)	E(Y0|t = 1)	KS	P	Control	Stop.method
1	Age	69.527	68.614	0.073	0.002	Used to smoke and still now	Unw
2	Edu	0.877	1.218	0.207	0.000	Used to smoke and still now	Unw
3	Marital	0.587	0.730	0.143	0.000	Used to smoke and still now	Unw
4	Gender	0.230	0.852	0.622	0.000	Used to smoke and still now	Unw
5	Living	0.246	0.145	0.101	0.000	Used to smoke and still now	Unw
6	Income	0.543	0.506	0.037	0.313	Used to smoke and still now	Unw
7	Work	0.183	0.183	0.000	1.000	Used to smoke and still now	Unw
8	Exercise	0.383	0.377	0.006	1.000	Used to smoke and still now	Unw
9	Illness	0.020	0.022	0.002	1.000	Used to smoke and still now	Unw
10	drink	1.733	1.039	0.381	0.000	Used to smoke and still now	Unw
11	Age	69.527	69.681	0.037	0.700	Used to smoke but not now	Unw
12	Edu	0.877	1.309	0.255	0.000	Used to smoke but not now	Unw
13	Marital	0.587	0.758	0.171	0.000	Used to smoke but not now	Unw
14	Gender	0.230	0.853	0.623	0.000	Used to smoke but not now	Unw
15	Living	0.246	0.270	0.024	0.983	Used to smoke but not now	Unw
16	Income	0.543	0.535	0.008	1.000	Used to smoke but not now	Unw
17	Work	0.183	0.247	0.063	0.117	Used to smoke but not now	Unw
18	Exercise	0.383	0.360	0.022	0.993	Used to smoke but not now	Unw
19	Illness	0.020	0.033	0.012	1.000	Used to smoke but not now	Unw
20	drink	1.733	1.093	0.394	0.000	Used to smoke but not now	Unw
21	Age	69.527	69.293	0.031	0.990	Used to smoke and still now	Es.mean
22	Edu	0.877	0.855	0.022	1.000	Used to smoke and still now	Es.mean
23	Marital	0.587	0.587	0.000	1.000	Used to smoke and still now	Es.mean
24	Gender	0.230	0.247	0.017	1.000	Used to smoke and still now	Es.mean
25	Living	0.246	0.199	0.046	0.779	Used to smoke and still now	Es.mean
26	Income	0.543	0.532	0.011	1.000	Used to smoke and still now	Es.mean
27	Work	0.183	0.173	0.010	1.000	Used to smoke and still now	Es.mean
28	Exercise	0.383	0.365	0.018	1.000	Used to smoke and still now	Es.mean
29	Illness	0.020	0.016	0.004	1.000	Used to smoke and still now	Es.mean
30	drink	1.733	1.715	0.011	1.000	Used to smoke and still now	Es.mean
31	Age	69.527	70.015	0.068	0.785	Used to smoke but not now	Es.mean
32	Edu	0.877	0.888	0.029	1.000	Used to smoke but not now	Es.mean
33	Marital	0.587	0.587	0.000	1.000	Used to smoke but not now	Es.mean
34	Gender	0.230	0.264	0.034	1.000	Used to smoke but not now	Es.mean
35	Living	0.246	0.215	0.031	1.000	Used to smoke but not now	Es.mean
36	Income	0.543	0.565	0.022	1.000	Used to smoke but not now	Es.mean
37	Work	0.183	0.184	0.000	1.000	Used to smoke but not now	Es.mean
38	Exercise	0.383	0.364	0.019	1.000	Used to smoke but not now	Es.mean
39	Illness	0.020	0.014	0.006	1.000	Used to smoke but not now	Es.mean
40	drink	1.733	1.727	0.010	1.000	Used to smoke but not now	Es.mean
41	Age	69.527	69.458	0.037	0.933	Used to smoke and still now	Ks.max
42	Edu	0.877	0.854	0.027	0.997	Used to smoke and still now	Ks.max
43	Marital	0.587	0.567	0.020	1.000	Used to smoke and still now	Ks.max
44	Gender	0.230	0.251	0.021	1.000	Used to smoke and still now	Ks.max
45	Living	0.246	0.209	0.037	0.934	Used to smoke and still now	Ks.max
46	Income	0.543	0.521	0.022	1.000	Used to smoke and still now	Ks.max
47	Work	0.183	0.192	0.009	1.000	Used to smoke and still now	Ks.max
48	Exercise	0.383	0.348	0.035	0.955	Used to smoke and still now	Ks.max
49	Illness	0.020	0.016	0.004	1.000	Used to smoke and still now	Ks.max
50	drink	1.733	1.704	0.015	1.000	Used to smoke and still now	Ks.max
51	Age	69.527	69.457	0.049	0.991	Used to smoke but not now	Ks.max
52	Edu	0.877	0.889	0.028	1.000	Used to smoke but not now	Ks.max
53	Marital	0.587	0.609	0.022	1.000	Used to smoke but not now	Ks.max
54	Gender	0.230	0.251	0.021	1.000	Used to smoke but not now	Ks.max
55	Living	0.246	0.205	0.041	0.999	Used to smoke but not now	Ks.max
56	Income	0.543	0.592	0.049	0.992	Used to smoke but not now	Ks.max
57	Work	0.183	0.171	0.012	1.000	Used to smoke but not now	Ks.max
58	Exercise	0.383	0.361	0.022	1.000	Used to smoke but not now	Ks.max
59	Illness	0.020	0.006	0.014	1.000	Used to smoke but not now	Ks.max
60	drink	1.733	1.777	0.022	1.000	Used to smoke but not now	Ks.max

Note: Unw represents unweighted method. Es.mean and Ks.max respectively use the effect size or the absolute standardized bias and summarizes across variables with the mean, and use the KS statistics to assess balances and summarizes using the maximum across variables

**Fig. 1:**
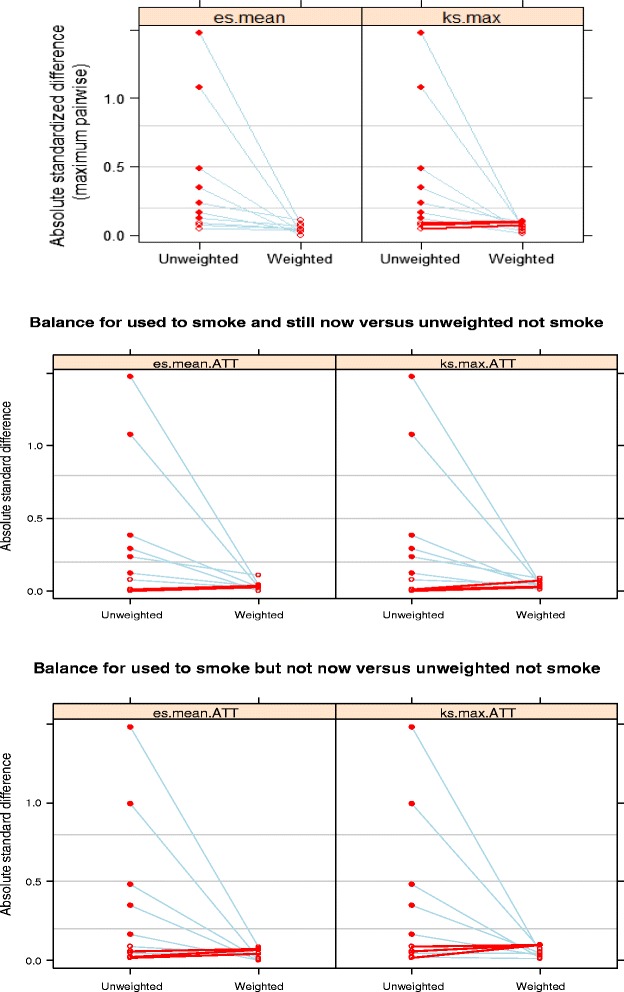
The standardized effect size plots before and after weighting

In order to demonstrate the importance and rationale of the PSM method in empirical research, useful diagnostic plots from propensity score objects were generated (see Fig. 1). Figure 1 is the standardised effect size plot illustrating the effect of weights on the magnitude of differences among groups on each covariate. The standardised effect size is defined as the treatment group mean minus the control group mean divided by the treatment group standard deviation. In these plots, closed circles indicate a statistically significant difference. Figure 1 shows many differences occurred before weighting, while none occurred after weighting. Furthermore, effect sizes of most variables were reduced after weighting (referring to the blue lines in Fig. 1).

### Estimation results

In non-randomized trials, the PSM method can maximize the elimination of sample selection bias and confounding bias [49]. As shown in Table 3, compared with non-smokers, the analysis estimated a decrease in self-evaluated memory of 0.0283 for smokers and an increase of 0.0054 for former smokers. For daily living activities, both groups decreased by 0.0735 and 0.0224, respectively. For cognitive function, the analysis estimated a 0.0091 decrease for smokers and a 0.0104 increase for former smokers. However, except for the effect on continued smokers in daily living activities, other effects did not appear to be statistically significant.

**Table 3:** Analysis of outcomes

Var1: Self-evaluation memory	Estimate	Std.Error	T value	Pr(>|t|)
Intercept	0.1478	0.0082	18.059	0.000^***^
Used to smoke and still now	−0.0283	0.0230	−1.232	0.218
Used to smoke but not now	0.0054	0.0383	0.141	0.888
Var2: Daily living abilities	Estimate	Std.Error	T value	Pr(>|t|)
Intercept	0.5529	0.0115	48.222	0.000^***^
Used to smoke and still now	−0.0735	0.0357	−2.057	0.034^*^
Used to smoke but not now	−0.0224	0.0531	−0.422	0.672
Var3: Cognitive function	Estimate	Std.Error	T value	Pr(>|t|)
Intercept	0.3721	0.0111	33.385	0.000^***^
Used to smoke and still now	−0.0091	0.0346	−0.262	0.793
Used to smoke but not now	0.0104	0.0525	0.198	0.843

Note: 10 % significance level; *5 % significance level; **1 % significance level; ***1 % significance level

## Discussion

Smoking is a widespread and serious issue in China. It has a certain sociality and function in social communication. However, smoking is harmful to people’s physical and mental health. Previous studies have indicated positive or negative expectations of smoking can affect smokers’ decisions, intentions and behaviours. Those with positive expectations of smoking believe smoking can promote social interaction, so most smoke and are not willing to give up the habit. Positive expectations can effectively predict the consequences of nicotine dependence [50]. Those who hold negative expectations of smoking worry about being rejected by peers if they smoke, so few smoke [51, 52].

This paper aimed to research whether smoking affects memory. The PSM was employed to reduce or eliminate confounding characteristics in observational data for smokers and non-smokers. PSM has gained widespread application over the past decade because of its advantages compared with traditional regression methods. PSM uses propensity score weights to control pre-treatment imbalances on observed covariates in non-randomised or observational data. After balancing or weighting, confounding characteristics are reduced or eliminated, and the distributions of observed pre-treatment characteristics are similar among the treated and controlled groups. This research study used propensity scores to weight the samples for the treatment group (non-smokers) and control group (current and former smokers).

Results showed that compared with non-smokers, current smokers decreased scores by 0.0283, 0.0735, 0.0091, respectively, on self-evaluated memory, daily living activities, and cognitive function. In contrast, former smokers decreased daily living activities by 0.0224, while they increased self-evaluated memory and cognitive function by 0.0054 and 0.0104, respectively. However, most effects did not appear to be statistically significant, except for the effect on daily living activities in current smokers. When people quit smoking, their self-evaluation and cognitive functions improve. Research results were generally insignificant in elderly Chinese samples. Possible causes include: (1) memory is affected by many factors, e.g. genetics, work demands, psychosomatic diseases, etc.; (2) this research used a three-level ordinal variable other than smoking history to measure smoking; (3) cognitive function related to coding in Chinese context. Although the education variable was added to adjust coding, respondent answers may also have had measurement error; and (4) the research sample consisted of elderly aged 60 and over, consequently, smoking may not have only related to their present life but also to their early childhood, adolescence, and adult lives.

Aging is becoming an increasingly serious problem in contemporary China. Due to smoking, elderly vulnerable groups bear significantly more health risks, which increases their children’s economic and personal care burdens [53, 54]. Furthermore, social burdens [55–59] and social pressure [60–62] also increse. Therefore, prohibiting campaign should be promoted.

## Conclusion

This study also had limitations: (1) Respondents were divided into three types: non-smokers, former smokers, and current smokers. In order to deeply mine the relationships between smoking and memory, future research could include a smoking frequency variable instead of a three-level variable. However, the current research lacked more detailed smoking indexes, e.g. the quality of cigarettes, combined index of the number of cigarettes and time. (2) The current study extracted three factors to denote memory: self-evaluated memory, daily living abilities, and cognitive function. However, these indexes were obtained through self-assessment, which may have had measurement error. In fact, accurately measuring memory is difficult. (3) The PSM only remove confounding by observed variables, that is, if there are some unmeasured variables differ among the treatment group and the control group, then the estimate may be biased. And the gender is very different between smokers and non-smokers in Table 1, perhaps gender is a main influence factor for memory loss. However, the PSM cannot reflect this difference. Therefore, in order to obtain more information on smoking and memory, further studies are needed.

## Footnotes

1 The 22 provinces include Liaoning, Jilin, Heilongjiang, Hebei, Beijing, Tianjin, Shanxi, Shannxi, Shanghai, Jiangsu, Zhejiang, Anhui, Fujian, Jiangxi, Shandong, Henan, Hubei, Hunan, Guangdong, Guangxi, Sichuan, and Chongqing.
